# Vertical Association Between Dietary Total Choline and L-alpha-glycerylphosphorylcholine and the Cognitive Function in Chinese Adults Aged over 55, Result from China Health and Nutrition Survey 1997–2018

**DOI:** 10.3390/nu16213713

**Published:** 2024-10-30

**Authors:** Fangxu Guan, Xiaofang Jia, Feifei Huang, Jiguo Zhang, Yanli Wei, Li Li, Jing Bai, Huijun Wang

**Affiliations:** 1 National Institute for Nutrition and Health, Chinese Center for Disease Control and Prevention, Beijing 100050, China; guanfx@ninh.chinacdc.cn (F.G.); jiaxf@ninh.chinacdc.cn (X.J.); huangff@ninh.chinacdc.cn (F.H.); zhangjg@ninh.chinacdc.cn (J.Z.); weiyl@ninh.chinacdc.cn (Y.W.); lili@ninh.chinacdc.cn (L.L.); baijing@ninh.chinacdc.cn (J.B.); 2 Key Laboratory of Public Nutrition and Health, National Health Commission of the People’s Republic of China, Beijing 100050, China

**Keywords:** choline, L-alpha-glycerylphosporylcholine, cognitive function, poor cognition

## Abstract

Background: With the aging process in China showing an accelerated trend, cognitive decline and impairment have become a major issue in older people. Dietary choline supplement may be a changeable lifestyle to improve this situation. Method: We analyzed 7659 adults aged over 55 in the China Health and Nutrition Survey (CHNS), evaluated cognitive function by the global cognition score, and found the association between cognitive function and dietary intake of total choline or L-alpha-glycerylphosphorylcholine (GPC). Linear and logistic mixed models with three levels were applied to analyze the association between dietary total choline/GPC intake and global cognition score, and the risk of poor cognition. Results: The average dietary intake at baseline was 178.8 mg/d for total choline, and 16.3 mg/d for GPC. After an average follow up of 6.8 years, we found that higher intake of total choline (β = 0.083, 95%CI: 0.046,0.119, *p* < 0.001) and GPC (β = 0.073, 95%CI: 0.034–0.111, *p* < 0.001) had positively associated with global cognitive scores. Additionally, higher intake of total choline had a better effect on improving the cognitive function of women (β = 0.092, 95%CI: 0.042–0.142, *p* < 0.01) and individuals between 55–65 years old (β = 0.089, 95%CI: 0.046–0.132, *p* < 0.01). However, higher GPC intake had a better effect on the cognitive function of men (β = 0.080, 95%CI: 0.020–0.141, *p* < 0.05). Higher total choline intake had a protective factor against poor cognition (OR = 0.762, 95%CI: 0.676,0.860, *p* < 0.001); the protective effect was more pronounced for women (OR = 0.750, 95%CI: 0.639,0.879, *p* < 0.001) and individuals aged 55–65 (OR = 0.734, 95%CI: 0.636–0.848, *p* < 0.001). Conclusions: higher dietary choline and GPC intake were beneficial for cognitive function, although we found that higher dietary choline was more effective in improving global cognitive scores at older ages; dietary choline should be supplemented as early as possible in old age to prevent poor cognition.

## 1. Introduction

Cognitive decline occurs with age in the middle-aged and elderly life stages, such as responsiveness and memory, language, visuospatial, and executive function abilities [1]. Mild cognitive impairment (MCI) may begin after midlife, but most often occurs at higher ages, which is the defining feature of Alzheimer’s disease (AD) and other dementias. It significantly increases the risk of functional dependence and poor quality of life in older people [2]. China’s aging process is quite rapid. According to the latest national census, more than 260 million people are over 60 years old, accounting for about 1/5 of the total population. China has the highest prevalence of dementia in the world, rising from 2.9% of people aged 60–69 to 31.9% of those over 90, putting a heavy burden on society and the health care system [3,4]. It is critical for screening and prevention of pre-dementia and pre-MCI among the Chinese population.

Choline is an essential nutrient for humans throughout their whole life and a main constituent of cell and organelle membranes. Choline participates in numerous physiological processes including DNA and histone methylation, signal transduction, and nerve myelination. Choline is a precursor of different metabolites including the neurotransmitter acetylcholine (ACh), the membrane phospholipids phosphatidylcholine (PC) and sphingomyelin, and the methyl donor betaine [5,6]. Humans can only produce small quantities of choline through the hepatic phosphatidylethanolamine N-methyltransferase pathway [7]; however, to avoid deficiency, most people need to gain choline from daily food [8,9].

Pre-clinical and clinical investigations have shown that GPC and other forms of choline supplementation have beneficial effects, especially in terms of improved cognitive performance [10,11]. There are many studies on the association between dietary total choline intake or blood choline concentration and cognition in the whole population, while longitudinal studies are mostly aimed at early life or children, and studies on adults and the elderly are mostly clinical trials [12]. Longitudinal research focusing on the older Chinese population must still be supplemented.

To address this question, we investigated the relationship between total choline/GPC intake and cognitive function through a dietary survey and cognitive screening of the elderly in a Chinese cohort.

## 2. Materials and Methods

### 2.1. Study Population

Our study was based on the China Health and Nutrition Survey (CHNS), a prospective household cohort study. The details of this cohort can be found in previous publications [13,14]. To date, the CHNS has already finished 12 rounds of surveys in 15 provinces/megacities including Beijing, Liaoning, Heilongjiang, Shanghai, Jiangsu, Zhejiang, Shandong, Henan, Hubei, Hunan, Guangxi, Guizhou, Chongqing, Yunnan, and Shannxi. Since 1997, CHNS has also collected cognitive information among older interviewers for six rounds of surveys. Thus, we selected people aged over 55 years old who participated in at least two complete surveys. We excluded those with abnormal total energy intake and abnormal choline intake. A total of 7659 individuals were enrolled in our analysis. All participants gave informed consent prior to the start of the investigation.

### 2.2. Cognitive Function Assessment

The cognitive screening items used in the CHNS included a subset of items from the Telephone Interview for Cognitive Status–Modified (TICS-m), which was investigated with face-to-face interviews [15]. TICS-m is a well-established screening tool for global cognitive function assessment in middle-aged and older people, and it is widely applied in cognitive function studies [16,17,18,19,20,21,22]. The TICS-m screening includes 3 modules. The first module is immediate and delayed recall of 10 words, with one point for each correct answer (score range 0–20); a total verbal memory score was constructed as the sum of the immediate and delayed 10-word recall. The second is counting backward from 20 to 1, 2 points for those who answer correctly the first time, 1 point for those who answer correctly the second time, and no points for those who answer more than twice or cannot answer (score range 0 to 2). The third module is serial 7 subtraction 5 times, with one point for each correct answer (score range 0–5). The global cognitive function score is the sum of these tasks (ranging from 0–27).

In our study, we chose the first quintile of the cognitive function test score as representing poor cognitive function, which corresponds to a global cognitive function score cutoff of <9.

### 2.3. Dietary Total Choline and GPC Assessment

We collected multiple sources of choline content in common foods in China. The total choline and GPC content of food were obtained based on the United States Department of Agriculture (USDA) database [23], China food composition tables [24], and the published literature [25,26,27], including free choline, phosphocholine, phosphatidylcholine, sphingomyelin, GPC, and betaine. The total choline content is the sum of free choline, phosphocholine, phosphatidylcholine, sphingomyelin, and GPC.

CHNS conducted household surveys for three consecutive days in each round, and all food intake of each participant was recorded over a three-day period. The total choline and GPC intake of each participant were calculated via our database and dietary survey. We quantified each food intake of each person within three days and then multiplied with the Choline/GPC content of each food.

### 2.4. Other Covariate Collection

Demographic indicators, smoking or alcohol consumption status, disease history, and physical activity were obtained via household questionnaires. Weight and height were measured through on-site investigation, and body mass index (BMI) divided weight by the square of height. The education level was divided into two groups, education below primary school and education of primary school or above. Smoking was grouped into non-smoking (never smoked and currently quitting smoking) and current smoking. Alcohol consumption was grouped into No alcohol in the past year and currently drinking. Income refers to the annual household income per capita, which was divided into tertiles: low-income, medium-income, and high-income groups.

Energy intake per day was calculated by energy-supplying nutrients of food intake multiplied by the energy coefficient.

The urbanization index was a validated 12-component score to capture the degree of urbanization in the communities. The scale included 12 domains with a total range of 0–120. The higher score indicates that there are more urban features across the various domains [28].

### 2.5. Statistical Analysis

We rescaled the global cognitive score into z-score by using baseline mean and standard deviation. All continuous independent variables are normalized. We divided the total choline and GPC intake of the population into three groups according to tertiles. The low total choline intake group with intake below 112 mg/d, medium: 112~205 mg/d, high: >205 mg/d. Low GPC intake group with intake below 8.69 mg/d, medium: 8.69~18.11 mg/d, high: >18.11 mg/d.

First, we created several empty models to find out if there was any necessity for a multi-level model. Then, we added the adjusted covariates in four consecutive times: model 1 with no adjustment, model 2 added sociodemographic variables, model 3 added lifestyle variables, and model 4 further added disease history and BMI.

We also conducted a hierarchical analysis to discover the effects of dietary total choline and GPC intake on cognitive function in different age groups (age between 55–65 at baseline and age > 65 at baseline) and different genders.

Multi-variate linear and logistic mixed models were applied in our study, using the GLLAMM module from STATA 15.0 to conduct the analysis [29]. The data cleaning and visualization used R 4.3.2.

## 3. Results

### 3.1. Baseline Characteristics

The mean follow-up time of 7659 respondents was 6.8 years. Among them, 4849 people participated in two rounds of the survey, accounting for 63.31%; 1062 people participated in three rounds, accounting for 13.87%; 1067 people participated in four rounds, accounting for 13.93%; 408 people participated in five rounds, accounting for 5.33%; and 273 people participated in all 6 rounds of the survey, accounting for 3.56%. Table 1 shows the baseline characteristics of our study population. The average dietary total choline intake of the population is 178.8 mg/d and GPC is 16.3 mg/d.

### 3.2. Vertical Association Between Total Choline and GPC Consumption and Cognitive Function

**Figure 1:**
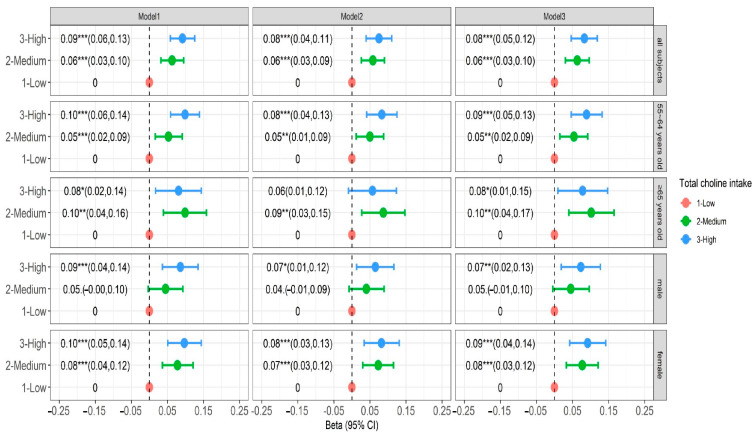
Vertical association between total choline intake and global cognitive score. Model 1 adjusts for age, gender, urban–rural, provincial, household per capita income, and education level (sociodemographic indicators). Model 2 adjusted smoking, alcohol consumption, total energy intake, and physical activity levels (lifestyle indicators) based on Model 1. Model 3 adjusted BMI and cardiovascular disease history (physical measurement and disease history indicators) based on Model 2. * *p* < 0.05, ** *p* < 0.01, *** *p* < 0.001.

Considering that our data is multi-level repeated measurement data, a three-level linear mixed model (community–individual–observation) was an optimal model for our analysis. As Model 3 showed, after adjusting all co-variates, medium and high levels of dietary total choline intake were positively correlated with global cognitive function z-score. A higher level of dietary total choline intake was associated with higher cognitive function scores. A high level of total choline consumption had a better effect on improving the cognitive function of females (β = 0.092, 95%CI: 0.042–0.142, *p* < 0.001) compared to males (β = 0.073, 95%CI: 0.019–0.127, *p* < 0.001), and had a better effect among individuals below 65 years old (β = 0.089, 95%CI: 0.046–0.132, *p* < 0.001) compare to individuals ≥65 years old (β = 0.078, 95%CI: 0.009–0.147, *p* < 0.05) (Figure 1).

**Figure 2:**
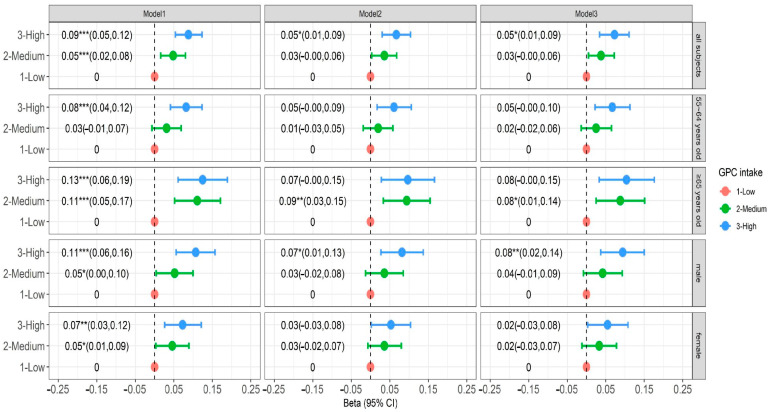
Vertical association between GPC intake and global cognitive score. Model 1 adjusts for age, gender, urban–rural, provincial, household per capita income, and education level (sociodemographic indicators). Model 2 adjusted dietary total choline intake, smoking, alcohol consumption, total energy intake, and physical activity levels (lifestyle indicators) based on Model 2. Model 3 adjusted BMI and cardiovascular disease history (physical measurement and disease history indicators) based on Model 2. * *p* < 0.05, ** *p* < 0.01, *** *p* < 0.001.

We also analyzed the global cognition z-score with dietary GPC intake. After adjusting total choline intake in the GPC model, a high level of dietary GPC intake was positively associated with higher global cognitive z-score (β = 0.052, 95%CI: 0.010–0.094, *p* < 0.05), and we found that higher GPC had significant effect cognitive function in men (β = 0.080, 95%CI: 0.020–0.141, *p* < 0.05) (Figure 2).

**Figure 3:**
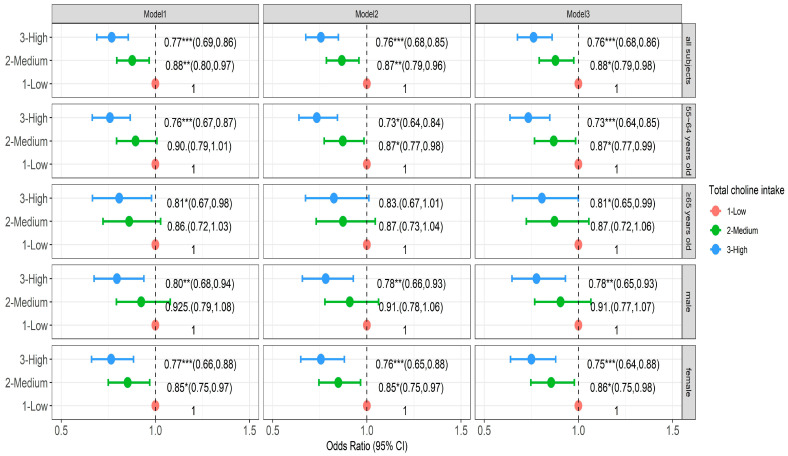
The effects (Odds Ratio) of dietary total choline intake on poor cognition. * *p* < 0.05, ** *p* < 0.01, *** *p* < 0.001. Model 1 adjusts for age, gender, urban–rural, provincial, household per capita income, and education level (sociodemographic indicators). Model 2 adjusted smoking, alcohol consumption, total energy intake, and physical activity levels (lifestyle indicators) based on Model 1. Model 3 adjusted BMI and cardiovascular disease history (physical measurement and disease history indicators) based on Model 2.

We also analyzed the effect of dietary total choline and GPC intake on poor cognition. Three-level logistic mixed models were applied. Model 3 showed that a high level of total choline consumption had a protective effect on the prevention of poor cognition (OR = 0.734, 95%CI: 0.676–0.860, *p* < 0.001), and a high level of total choline consumption had a more obvious protective effect on poor cognition in females (OR = 0.750, 95%CI: 0.639–0.879, *p* < 0.001) than in males (OR = 0.777, 95%CI: 0.647–0.931, *p* < 0.01), and high levels of total choline consumption in older people under 65 years old has a more protective effect (OR = 0.734, 95%CI: 0.636–0.848, *p* < 0.001) than individuals aged over 65 years old (OR = 0.806, 95%CI: 0.650–0.999, *p* < 0.05) (Figure 3). However, we did not find the effect of dietary GPC on poor cognition.

## 4. Discussion

Our study examined the effect of dietary total choline and GPC consumption on cognitive function in Chinese adults aged ≥ 55 years old, and analyzed the combined data of six rounds of CHNS datasets from 1997 to 2018. As our results show, people in the high dietary total choline (>205 mg/d) group and high GPC (>18.11 mg/d) intake group had higher global cognitive scores. The results indicated that the dietary intake of total choline and GPC were beneficial for cognitive function. High dietary total choline and GPC intake were more effective in improving cognitive function in older people. However, people should eat more choline-rich foods at a younger age because higher choline intake has a more protective effect against poor cognition in old age.

Pre-clinical studies have shown that low plasma free choline concentrations were associated with poor cognitive performance, high choline (>8.4 μmol/L) concentration was associated with better test scores in the Trail Making Test part A, modified versions of the Digit Symbol Test, and the Mini-Mental State Examination [30]. In a community-based population of normal mental status individuals, compared with low choline consumption, higher choline consumption was related to better cognitive performance [31]. Study results from the Framingham Heart Study Offspring Cohort show that low choline intake (defined as ≤219 and ≤215 mg/d for dementia and AD, respectively) was found to be significantly associated with incident dementia and incident AD [32]. However, data from the U.S. National Health and Nutrition Examination Survey 2011–2012 and 2013–2014 study did not find a significant connection between dietary total choline intake and cognitive functioning in older U.S. adults [10]. Random clinical trials of choline intervention have also reported that both total choline and GPC intakes were associated with better performance in cognitive tests assessing frontal and temporal lobe functioning. Higher total choline intake was associated with better performance in verbal fluency and memory functions. Higher GPC intake was related to a lower risk of having dementia and better cognitive performance in men in eastern Finland [33].

Many studies have shown that choline, GPC, and other methyl donors play a beneficial role in normal neurodevelopment and neurocognitive function. They are considered to be neuroprotective agents, regulating the expression of key genes related to memory, learning, and cognitive function through epigenetic mechanisms [34]. This is not only critical for normal growth and function of the early brain, but also has good effects in enhancing brain function, delaying or alleviating cognitive decline with age, and neurodegenerative diseases such as AD [12].

According to our study, the average daily dietary choline intake was 191.0 mg/d for men and 167.7 mg/d for women. The Dietary Reference Intakes for China (2023) indicated that the AI of dietary choline for adults is 500 mg/d for men and 400 mg/d for women [35]. Our findings suggested that the dietary total choline intake among Chinese older people is lower than the AI, and the dietary total choline intake of older people in China needs to increase. For Chinese people, especially the elderly, daily micronutrient intake is still a major problem. Choline and its derivatives are widely distributed in eggs, fish, and milk, as well as cruciferous vegetables, soybeans, etc. [12,27]. People should choose more of these foods to intake each kind of choline and other essential micronutrients.

In this study, we were able to evaluate dietary choline and GPC intake of Chinese residents with an integrating database, then conducted a longitudinal analysis of Chinese older people and found clues to the association between dietary total choline/GPC intake and cognitive function. However, there is little data on choline in Chinese food compositions, and because there are differences in common food compositions from the U.S., we may underestimate the intake level of choline by Chinese residents. Further measurement of choline composition in common Chinese foods is needed to fill the blank in the Chinese dietary choline database.

## 5. Conclusions

In conclusion, higher dietary total choline and GPC intake were beneficial for cognitive function. Although we found that higher dietary total choline and GPC were beneficial in improving global cognitive scores at older ages, dietary total choline should be supplemented as early as possible in old age to prevent poor cognition, and we have not yet found the preventive effect of GPC on poor cognition. The awareness of the importance of choline among Chinese residents should be raised, and dietary choline intake from daily food should be appropriately increased.

## Figures and Tables

**Table 1:** Baseline characteristics of the study population.

Characteristics	Baseline Age < 65	Baseline Age ≥ 65	Males	Females	Total
N	5394	2265	3661	3998	7659
Baseline age, mean ± SD (years)	59.2(2.8)	71.4(5.3)	62.6(6.6)	63.0(6.8)	62.8(6.7)
Follow-up years, mean ± SD	7.4(6.0)	5.5(4.5)	6.7(5.6)	6.9(5.8)	6.8(5.7)
Urban residents, No (%)	1955(36.2)	1212(53.5)	1463(40.0)	1704(42.6)	3167(41.4)
Education below primary school, No (%)	2934(54.4)	1427(63.0)	1722(47.0)	2639(66.0)	4361(56.9)
Per capita income, median(P_25_,P_75_) (yuan)	5789.2(2263.3, 17,196.3)	6114.0(1821.9, 22,393.2)	6106.3(2195.8,18,460.7)	5612.7(2104.0, 18,782.8)	5861.7(2148.9, 18,658.6)
Urbanization index, mean ± SD	65.7(19.8)	69.9(19.9)	66.5(19.9)	67.3(19.9)	66.9(19.9)
Currently smoke, No (%)	1709(31.7)	605(26.7)	2113(57.7)	201(5.0)	2314(30.2)
Alcohol use, No (%)					
None	3674(68.9)	1669(74.3)	1694(46.7)	3649(92.3)	5343(70.4)
2 times/wk or less	448(8.4)	150(6.7)	467(12.9)	131(3.3)	598(7.9)
3 or more times/wk	1211(22.7)	427(19.0)	1465(40.4)	173(4.4)	1638(21.6)
Total energy intake, mean ± SD (kcal/d)	2194.7(752.2)	1963.0(683.3)	2299.4(768.7)	1967.6(675.1)	2126.2(740.1)
Physical activity, median(P_25_,P_75_) (METS/wk)	115.9(47.6, 262.1)	47.5(15.9, 105.8)	85.0(23.1, 232.5)	87.9(43.9, 210.9)	86.8(35.5, 222.2)
Body Mass Index, mean ± SD (kg/m^2^)	23.9(3.7)	23.5(3.7)	23.5(3.4)	24.0(3.9)	23.8(3.7)
Cardiovascular disease history, No (%)	812(15.1)	497(21.9)	465(18.2)	644(16.1)	1309(17.0)
Total choline intake, median(P_25_,P_75_) (mg/d)	159.0(93.4, 238.9)	152.0(90.8, 240.3)	168.1(99.7, 252.8)	146.5(86.9, 227.1)	156.1(92.7, 239.4)
GPC intake, median(P_25_,P_75_) (mg/d)	13.3(7.4, 22.4)	13.0(7.1, 21.2)	14.5(8.2, 23.6)	12.2(6.5, 20.3)	13.2(7.3, 22.0)

## Data Availability

Data is available by contacting the corresponding author for reasonable request.
